# Safety and efficacy of extended thrombophilia screening directed venous thromboembolic events (VTE) prophylaxis in live liver donors: do we really need extended thrombophilia screening routinely?

**DOI:** 10.1097/MS9.0000000000001772

**Published:** 2024-01-30

**Authors:** Abdul Wahab Dogar, Azhar Hussain, Kaleem Ullah, Shams-ud-din, Abdul Ghaffar, Khabab Abbasher Hussien Mohamed Ahmed, Muhammad Junaid Tahir

**Affiliations:** a Pir Abdul Qadir Shah Jeelani Institute of Medical Sciences, Gambat, Sindh; b Pakistan Kidney and Liver Institute and Research Centre (PKLI & RC), Lahore, Pakistan; c Faculty of Medicine, University of Khartoum, Khartoum, Sudan

**Keywords:** live liver donors, living donors liver transplantation (LDLT), thrombophilia screening, venous thromboembolic events

## Abstract

**Background and aims:**

The study aimed to determine the prevalence of hereditary thrombophilia, and stratify its severity among live liver donors in Pakistan. Also, the authors evaluated the safety and efficacy of thrombophilia profile testing directed venous thromboembolic events (VTE) prophylaxis while balancing bleeding risk and the need for routine thrombophilia testing before live liver donation among living donor candidates.

**Materials and methods:**

Protein S (PS), protein C (PC), anti-thrombin (AT) III, and anti-phospholipid antibody panel (APLA) levels were measured in 567 potential donor candidates. Donors were divided into normal, borderline and high-risk groups based on Caprini score. The safety endpoints were VTE occurrence, bleeding complications or mortality.

**Results:**

Among 567 donors, 21 (3.7%) were deficient in protein C, and 14 (2.5%) were deficient in anti-thrombin-III. IgM and IgG. Anti-phospholipids antibodies were positive in 2/567 (0.4%) and 2/567 (0.4%), respectively. IgM and IgG lupus anticoagulant antibodies were positive in 3/567 (0.5%) and 3/567 (0.5%), respectively. VTE events, bleeding complications and postoperative living donors liver transplantation-related complications were comparable among the three donor groups (*P*>0.05). One donor in the normal donor group developed pulmonary embolism, but none of the donors in either borderline or high-risk group developed VTE. The mean length of ICU and total hospital stay were comparable. No donor mortality was observed in all donor groups.

**Conclusions:**

Due to thrombophilia testing directed VTE prophylaxis, VTE events were comparable in normal, borderline and high-risk thrombophilia donor groups, but more evaluations are required to determine the lower safe levels for various thrombophilia parameters including PC, PS and AT-III before surgery among living donor candidates.

## Background

Highlights

- In addition to reporting safety and venous thromboembolism (VTE) prophylactic methods in living liver donors (LLDs), the purpose of this study was to ascertain the prevalence of inherited thrombophilia in the Pakistani community.
- Between 1 March 2020 and 28 February 2022, 567 potential living liver donors underwent thrombophilia testing that included anti-thrombin (AT) III, protein S (PS), anti-phospholipid antibody (APLA), and protein C (PC). The donors in this retrospective analysis were split into three groups: normal, borderline, and high-risk. VTE incidence, bleeding complications, or fatality were the safety goals.
- VTE events, bleeding complications and postoperative LDLT-related complications were comparable among the three donor groups. One donor in the normal donor group developed pulmonary embolism, but none of the donors in either borderline or high-risk group developed VTE.
- In conclusion, donor surgery is safe for donors who fall into either the borderline or high-risk categories. However, additional research is needed to establish the necessity and lowest safe levels of PC, PS, and AT-III of thrombophilia testing among living donor candidates.

Vascular events after living donor liver transplantation (LDLT) are one of the most serious complications that can lead to significant donor and recipient morbidity and mortality. Recipient-related vascular thromboembolic complications are well studied and recognized as a significant cause of graft loss and mortality, especially early hepatic artery thrombosis (eHAT)^1, 2^. However, venous thromboembolic complications are not extensively studied in live liver donors and there is a variation in donor screening and venous thromboembolic event (VTE) prophylaxis protocols among LDLT centres.

As the highest priority in living donor liver transplantation is donor safety, so all potential risk factors that may lead to donor morbidity and mortality are ruled out before pursuing donor hepatectomy. One such risk factor is hereditary thrombophilia profile status which may cause significant VTE events post-hepatectomy in donors. One LDLT centre from Japan had reported a case of a donor who developed pulmonary embolism (PE) in the early postoperative period despite no obvious risk factors of thrombosis by normal preoperative evaluation of the donor. Subsequent evaluation revealed that protein S (PS) activity was low in that donor^3^. After this, the majority of the LDLT centres started performing screening tests for hereditary thrombophilia along with conventional coagulation tests for the investigation of donors and determined their VTE prophylaxis criteria accordingly^1^.

As per our centre’s donor standard evaluation protocol, every donor underwent extended hereditary thrombophilia testing including PS, PC, and AT-III activity, and levels of lupus anticoagulant, IgG anti-CL antibodies, IgM anti-CL antibodies, and anti-B2 GPI antibodies along with conventional coagulation parameters. To add to the current literature on venous thromboembolic complications in live liver donors post-hepatectomy in LDLT, this study was conducted to determine the safety and efficacy of thrombophilia profile testing directed VTE prophylaxis while balancing for bleeding risk. Also, we tried explaining whether thrombophilia profile testing should be done routinely before live liver donation among living donor candidates before LDLT. We hope this experience will better inform screening decisions, and motivate discourse regarding future standardization of practice.

## Material and methods

### Study setting and design

This prospective cohort study was conducted at the organ transplant unit at Pir Abdul Qadir Shah Jeelani Institute of Medical Sciences (PAQSJIMS), Gambat, Pakistan, from 1 March 2020 to 28 February 2022. During the study period, 585 potential live liver donors for LDLT were evaluated at our centre. Fourteen donors were excluded from the study due to recipient death or technical delisting. This study was registered by the institutional ethical review board of the PAQSJ Institute of Medical Sciences, Gambat, Pakistan (Reference UIN: PAQSJIMS/IRB/865).

### Donor selection criteria

Live liver donors (LLDs) were selected after a detailed interview, physical health examination (PHE), and laboratory investigations (Table 1). We also performed the psychological assessment, coercion, and self-voluntariness for candidacy after sharing the details of the surgical procedure, risks of complications (10–15%), and a 0.5% chance of mortality. Criteria used for donor selection include age between 18 and 40 years, BMI less than 30 kg/m^2^, Landsteiner’s ABO blood grouping compatibility, absence of comorbid conditions such as uncontrolled hypertension, hyperlipidemia, diabetes mellitus, ischaemic heart disease, and chronic respiratory diseases. We ensured that all the laboratory parameters were normal. We accepted Liver attenuation index (LAI) greater than 1, functional liver remnant (FLR) greater than 30%, graft-to-recipient weight ratio (GRWR) greater than or equal to 0.7 and acceptable vascular anatomy based on triphasic computerized tomography (CT) scan. Magnetic resonance cholangiopancreatography (MRCP) we performed to delineate the biliary tree anatomy^4^.

**Table 1:** Live liver donor evaluation process.

Step1
• Blood tests: Grouping, complete blood count, prothrombin time/international normalized ratio, liver function tests, urea, glucose, albumin, creatinine, magnesium, electrolytes, urine R/E, HCV antibody, hepatitis B profile (hepatitis B surface antigen, hepatitis B core antibody, hepatitis B surface antibody), human immunodeficiency virus 1 and 2 screen • Radiology: Chest X-ray PA view • Cardiology: Electrocardiogram • Consultation: Transplant surgeon
STEP 2
• Radiology: Liver dynamic CT scan • Radiology: MRCP,
STEP 3
• Echo 2D with pulmonary pressure readings • Extended Thrombophilia screen: Anti-thrombin III, protein S, protein C, resistance V (factor V), factor II, Lupus anticoagulant (IgG & IgM), anti-cardiolipin (IgG & IgM), anti-b2GPI (IgG & IgM) • Biochemistry: G6PD, reticulocyte count, sickle cell, haemoglobin A1c, lipid profile, thyroid function tests, serum ferritin, ceruloplasmin, alpha-1 antitrypsin • Immunology: IgA, IgG, IgM, ANCA, anti-nuclear antibody group (anti-nuclear antibody, anti–smooth muscle actin, anti-mitochondrial antibody), antibody screen, cytomegalovirus IgG • Consultations: Psychiatrist, anaesthetist, hepatologist, independent assessor

2D, two dimensional; CT, computed tomography; G6PD, glucose 6 phosphate dehydrogenase; HCV, hepatitis C virus; MRCP, magnetic resonance cholangiopancreaticography.

We rejected LLDs unwilling to donate at any stage of their evaluation, those with deranged laboratory parameters and FLR less than 30%, portal vein-type D and E, segment IV supplied by right hepatic artery intra-hepatically (on CT scan), and donors having more than 2 ducts (on MRCP). We also rejected donors with GRWR less than 0.70. WHO Class I G6PD deficiency was considered a contraindication^5^.

All donors were assessed by an independent physician not related to the transplant team to avoid the influence of the liver surgical team. After completion of the evaluation, triple consent (informed verbal, written and video consent) was obtained from the donors with an understanding of the voluntary nature of donation and backing out at any time before surgery. Following donor selection, approval from the Human Organ Transplant Authority (HOTA), Pakistan, was sought before donation. According to HOTA, only blood-related donors were selected for liver donation.

### Extended hereditary thrombophilia screening testing

Protein S (PS), protein C (PC), anti-thrombin (AT) III, anti-phospholipid antibody (APLA) and lupus anticoagulant antibody levels were measured in 567 potential donor candidates. Information obtained from the history of thrombosis and conventional coagulation tests, including PT, aPTT, serum fibrinogen, and D-dimer levels, was also evaluated. The donor candidates were then grouped according to the thrombophilia screening results score.

Donor candidates who met all of the criteria for normal levels of PS, PC, factor II, AT-III, [PS >60%, PC (69.1–140%), AT-III (75.1–125%)] and APLA negative (lupus anticoagulant LA1/LA2<1.2 s), IgG anti-CL antibodies less than 20 U/ml, IgM anti-CL antibodies less than 13 U/ml, IgG anti-b2GPI less than 5 U/ml and IgM anti-b2GPI less than 5 U/ml were classified in the normal group^3^. All donor candidates other than those in the normal group were placed in the suspected group. After 400 LDLT, factor II was discarded from routine donor evaluation after haematological consultation.

Thrombophilia screening tests were repeated after 4–6 weeks from initial testing in the suspected group to counter abnormal assay results and thus, prevent misdiagnoses^6–9^. After initial and secondary tests, potential candidates in the suspected group were referred to haematologists. After discussion with haematologists, donors in the suspected group were eventually subdivided into the borderline and high-risk groups according to the thrombophilia screening results based on the Caprini score^10^ (Table 2). Donor candidates with a score of 1–2 were put in the borderline group, while donors with 3–4 scores were put in the high-risk group. Donors in borderline and high-risk had a Doppler ultrasonography (USG) of the lower limbs to rule out any preexisting DVT before the surgery.

**Table 2:** Thrombophilia screening test scoring.

Variables	Score=0	Score=1	Score=2	Score=3
Protein S^a^ (%)	>60	50–60	40–50	<40
Protein C^a^ (%)	>64	54–64	44–54	<44
Anti-thrombin III (%)	>70	60–70	50–60	<50
LAC^b^	<1.3	>1.3	—	—
IgG anti-cardiolipin antibodies (U/ml)	<10	>10	—	—
Anti-beta 2 glycoprotein antibodies	<3.5	>3.5	—	—
Medical history of thrombosis	No	Yes	—	—

LAC, indicates Lupus Anticoagulants.

### Strategy of prophylaxis against thrombosis

For prophylaxis of VTE in donors in the normal group, we followed the CHEST/ACCP guidelines based on VTE prophylaxis and management^11^. Following surgery, elastic stockings and intermittent pneumatic compressions (IPCs) were used in all donor groups, and early ambulation was encouraged. This was supplemented with low molecular heparin in the borderline and high-risk groups for prophylaxis against VTE^11^. The median duration of heparin administration for donors in the borderline and high-risk group was 5 days (range, 1–7 days).

### Postoperative monitoring for thrombosis

Symptoms related to thromboses, vital signs, and oxygen saturation were carefully monitored postoperatively. When thrombosis was suspected, further evaluation was done which included chest X-ray, Doppler USG of lower limbs, contrast-enhanced chest CT scan, and arterial blood gas levels analysis, as required.

### Statistical analysis

We stored and sorted our donors’ data in a centralized data repository at our centre, prospectively. IBM SPSS Statistics software version 25 (IBM Corp) was used to analyze data. All the quantitative values were given as the mean ±SD and qualitative variables were given as frequencies/percentages (%). Qualitative variables were compared between the study groups using the χ^2^ test/Fischer Exact test to test the statistical significance. An independent *t*-test was used to compare the means of various quantitative variables. *P* less than 0.05 was considered to be statistically significant.

The work has been reported in accordance with STROCSS guidelines^12^.

## Results

### Demographics

We evaluated 585 live liver donors for LDLT at our centre. Fourteen donors were excluded from the study due to recipient death or technical delisting. Five hundred sixty-seven donors consisting of 318 (56.08%) females and 249 (43.92%) males with a mean age of 24.73± 5.54 years were included in statistical analysis (Table 3).

**Table 3:** Comparison of demographics and surgical features of LLDs among donor groups.

Variables	Normal group (*n*=516)	Borderline group (*n*=44)	High-risk group (*n*=7)	*P*
Mean age (years)	23.43±5.53	23.91± 5.25	26.86±6.54	0.17
Mean BMI (Kg/m^2^)	21.40±7.99	20.5±2.71	21.53±3.29	0.69
Sex, *n* (%)				
Male	290 (56.2)	24 (54.5)	4 (57.1)	0.97
Female	226 (43.7)	20 (45.4)	3 (42.8)	
Marital status, *n* (%)				
Unmarried	351 (68.1)	25 (56.9)	5 (71.4)	0.06
Married	165 (31.9)	19 (43.1)	2 (28.5)	
Donors relation to recipients, *n* (%)				
Son	63 (12.2)	6 (13.6)	1 (14.2)	0.008
Brother	99 (19.1)	5 (11.3)	2 (28.5)	
Nephew	25 (4.8)	5 (11.3)	1 (14.2)	
Daughter	35 (6.7)	5 (11.3)	0	
Sister	32 (6.2)	5 (11.3)	0	
Father	88 (17.1)	3 (6.8)	0	
Swap	10 (1.9)	0	0	
Others	157 (30.4)	11 (25.0)	3 (42.8)	
Type of graft, *n* (%)				
Modified right lobe graft	437 (84.6)	36 (81.8)	6 (85.7)	NS
Modified extended right lobe	62 (12.0)	8 (18.1)	1 (14.2)	
graft	4 (0.7)	7 (15.9)		
Left lobe graft	11 (2.1)	0	0	
Left lateral segment graft	6 (1.1)	0	0	
GRWR	1.26±0.63	1.4±0.68	1.2±0.63	NS
Mean warm ischaemia time (min)	10.99±6.09	10.68±4.08	13.86±7.26	
Mean operation time (h)	407.94±64.86	412.27±89.11	422.86±62.37	
Mean blood loss (ml)				NS
Blood transfusions (no. patients/%)	516	44	7	0.76
Personal history of thrombosis	0	0	0	—
Family history of thrombosis	0	0	1	—

GRWR, graft-to-recipient weight ratio; LLD, living liver donor; NS, not specified.

### Extended thrombophilia screening testing results and donor classification

Out of 576 donors, 509 subjects were classified into the normal group and 63 donors in the suspected group on their first thrombophilia screening testing results. On repeat extended thrombophilia testing, 7 donors were relocated to the normal group, making a total of 516 donors in the normal donors’ group. Donors in the suspected group were sent to a haematologist for further evaluation. Among them, 21 (3.7%) donors were deficient in protein C, 14 (2.5%) were deficient in anti-thrombin-III on repeat testing. IgM and IgG anti-phospholipids antibodies were positive in 2/567 (0.4%) and 2/567 (0.4%), respectively. IgM and IgG lupus anticoagulant antibodies were positive in 3/567 (0.5%) and 3/567 (0.5%), respectively.

According to haematology consultation and Caprini score, donors in the suspected group were grouped under the borderline group (*n*=44) and high-risk group (*n*=12). 5 donors were excluded as their both PC (%) and Anti-thrombin-III (%) activity were less than 50%. We kept 7 donors in the high-risk group whose were having their both PC (%) and anti-thrombin-III (%) activity of more than 50% but below the normal range. There were no differences in demographics and various conventional donor parameters among various donor groups (*P*>0.05) (Table 3).

### Outcomes

#### Complications among LLDs in the normal donors group

In the normal donor group, 454 (88.0%) donors were discharged from the hospital uneventfully without any complication as compared to 29 (65.9%) in the borderline group and 5 (71.5%) in the high-risk group, *p* value=0.43). 31 (6.0%) and 31 (6.0%) donors in normal group developed Clavien–Dindo grade 1 and 2 and grade 3 and 4a complications respectively. Out of those donors who developed grade 3 and 4a complications in the normal group, 09 (1.7%) required thoracentesis for symptomatic pleural effusion, 06 (1.1%) required abdominal drains for collections and biloma, 3 (0.5%) required ERCP with biliary stenting due to biliary leakage. Reoperation during the postoperative period was needed in 03 (0.5%) donors for hemoperitoneum due to perihilar region bleeding. 1(0.19%) donor in the normal group had a pulmonary embolism during ICU stay and was re-intubated. All these complications were managed successfully and no mortality was observed (Table 4).

**Table 4:** Various outcomes in LLDs in donor groups according to Clavien–Dindo classification system.

Variable	Normal group (*n*=516)	Borderline group (*n*=44)	High-risk group (*n*=7)	*P*
Total no. complications, *n* (%)	62 (12.0)	15 (34.1)	2 (28.5)	0.43
Grade 1 and 2, *n* (%)				
Wound infections	20 (3.8)	3 (6.8)	2 (28.5)	0.001
Wound haematoma	2 (0.3)	1 (2.2)	0	
UTI	4 (0.7)	2 (4.5)	0	
Fever	3 (0.5)	2 (4.5)	0	
Paralytic ileus	2 (0.3)	1 (2.2)	0	
Grade 3A, *n* (%)				
Bile leakage	6 (1.1)	1 (2.2)	0	0.15
Bile duct stricture	3 (0.5)	0	0	
Postoperative bleeding	3 (0.5)	1 (2.2)	0	
Postoperative VTE event	1 (0.1)	0	0	
Pleural effusion/Aspiration	9 (1.7)	1 (2.2)	0	
ERCP and stenting	3 (0.5)	1 (2.2)	0	
Grade 3B, *n* (%)				
Re-open	6 (1.1)	1 (2.2)	0	0.45
Grade 4A, *n* (%)				
Need ICU care/ventilator	1 (0.1)	1 (2.2)	0	0.95
Grade 4B	0	0	0	—
Multi-organ failure				
Grade 5	0	0	0	—
Mean ICU stay (days)	2±1	2.53±1	3±1	0.15
Mean hospital stay (days)	6±2	5±2	6±2	0.17
Mortality	0	0	0	—

ERCP, endoscopic retrograde cholangiopancreaticography; LLD, living liver donor; UTI, urinary tract infection; VTE, venous thromboembolic event.

#### Complications among LLDs in borderline and high-risk donors group

09 (20.45%) and 06 (13.63%) donors in the borderline group developed Clavien–Dindo grade 1 and 2 and grade 3 and 4a complications, respectively, while 02 (28.5%) donors in the high-risk group developed Clavien–Dindo grade 1 and 2 complications. Out of those donors who developed grade 3 and 4a complications in the borderline donor group, 01(2.2%) required thoracentesis for sympathetic pleural effusion, 01 (2.2%) required abdominal drains for collections and biloma, 01 (2.2%) required ERCP with biliary stenting due to biliary leakage. None of the donors in either borderline or high-risk donor group developed any VTE-related complications. There was no statistically significant difference in other postoperative complications between the donors groups (*P*>0.05) (Table 4).

#### Healthcare utilization (ICU stay and total length of hospital stay)

Mean ICU stay was 2±1 day in normal and high-risk groups as compared to 2.53±1 day in borderline group, respectively, *p* value=0.15. Similarly, the mean total length of hospital stay was 6±2 days in normal & high-risk groups and 5±2 days in borderline group, *p* value=0.17 (Table 4).

#### Mortality

There was no donor mortality observed during our study (Table 4).

## Discussion

In this prospective cohort study, we evaluated the safety and validity of our algorithm for donor selection and strategy of prophylaxis of postoperative VTE events according to the extended thrombophilia screening tests results. Fifty-one living donors having borderline or high-risk VTE Caprini scores for extended thrombophilia screening tests were given low molecular weight heparin postoperatively to decrease the risk of VTE. As a result of this strategy, none of the donors developed VTE in our study. This is the first report to determine the importance of risk stratification approaches for VTE prophylaxis based on extended thrombophilia screening in live liver donors in a large cohort including high VTE risk donors to the best of our knowledge. None of the previously published studies evaluated the risk of VTE and other complications in high-risk donors as per the extended thrombophilia testing and Caprini score.

No evidence supports universal thrombophilia screening tests in patients who are undergoing major surgery^13^. However, in the case of living donors, evaluation and management require more caution because the safety of the donor is the highest priority in LDLT. Advanced age, obesity, myocardial infarction, and hormonal therapy are the risk factors for VTE^14^. As most of these risk factors are usually removed in the process of donor evaluation and selection, hereditary thrombophilia such as deficiency of PC, PS, AT-III, and lupus anticoagulants are also risk factors for VTE^14^ in the healthy population. So, as per our centre protocol, we performed extended thrombophilia testing on all the potential donor candidates. Among others, the most significant risk factor for VTE occurrence is a previous history of thrombosis, which was also investigated during donor evaluation. However, many thrombophilia cases are latent, and thrombosis can be triggered by various stressors like pregnancy or surgery^15^. The majority of the Pakistani healthcare centres do not have access to electronic health record (EHR) or electronic medical record (EMR) systems for healthcare-related information storage and practice management and even if they have some degree of EMR, they are not inter-linked and cannot be accessed by other centres. In this situation, asking donor candidates about their previous medical history of thrombosis has some importance, but it is not enough to rule out donor candidates with hereditary thrombophilia and subsequently, prevent VTE occurrence in the immediate post-hepatectomy period.

Unfortunately, 63 out of 567 potential donors tested positive for one or more components of extended thrombophilia screening after their initial screening at our centre. In this study, results from the standard coagulation tests, such as platelet count, PT, and aPTT, did not distinguish between donor candidates in the normal and thrombophilia donors. These findings demonstrate that standard screening criteria are insufficient for accurately determining thrombophilia. So, due to the lack of medical history for VTE events and non-reliability of the standard coagulation tests, hereditary thrombophilia testing is essential and important to screen donor candidates for hereditary thrombophilia, as recommended by other studies^15–17^. Also, five donor candidates were rejected for donation as both PC (%) and anti-thrombin-III (%) activity were less than 50% and there was an increased likelihood of VTE development in these donors. After haematology consultation, we thought that their thrombophilia screening results were serious enough to exclude them from the donor pool because it was reported that severely thrombophilic patients can develop VTE after surgery, even in the setting of the use of anticoagulants^16^. Although these donor candidates might not have developed VTE after surgery we believed that excluding these candidates was reasonable to keep donor safety at the highest level. There is no consensus on the minimum cut-off level of PS or PC because of the existence of large standard deviations^18^. That is why, the minimum normal values for determination of risk of VTE development cannot be determined clearly. However, more studies are required to clearly define the lowest cut-off level of PS and PC safe for LDLT without compromising the safety of donors.

Also, 31/416 (7.45%) were deficient in factor II which was a significantly higher ratio and was bringing logistical, and financial challenges to our centre and recipients’ families. In the wake of the strict donation criterion of selecting only blood-related donors by the Human Organ Transplant Authority (HOTA), Pakistan and almost non-existent deceased donor liver donation, this factor-II deficiency was causing exhaustion of the already dwindling liver graft pool. Due to these challenges, we discontinued testing for factor-II deficiency after 416 LDLTs after a consensus from a multidisciplinary meeting involving liver transplant surgeons, transplant hepatologists, haematologists and transplant anaesthetists. Also, the anti-phospholipid antibodies panel (lupus anticoagulant, IgG and IgM anti-CL antibodies and IgG and IgM anti-b2GPI) was positive in a very small fraction of donors (0.5%) in a titre that was too low to be clinically significant. For the detection of hereditary thrombophilia, PS and PC activity are good indicators^9^. We recommend that discontinuation of routine anti-phospholipid antibodies panel is reasonable, especially if other hereditary thrombophilia tests including protein C and protein S are negative. We also recommend against extended thrombophilia screening in routine donor evaluation as it does not alter outcomes brings more financial burden and shortens the donor pool. Based on our centre experience, we recommend limited and targeted thrombophilia screening with Protein S (PS), protein C (PC), and anti-thrombin (AT) III during routine donor evaluation. Further, extended thrombophilia screening can be carried out if the initial limited and targeted screen returns positive. Given the current shortage of donors, especially in those areas in which deceased donor grafts have less supply, this strategy will maximize our chances of increasing the donor pool and graft supply without compromising donor safety.

An elevated risk of VTE has been linked to liver resection due to a postoperative hypercoagulable state. This hypercoagulability can be partially explained as PC and AT-III levels were shown to be much lower after liver resection than after major abdominal surgeries^19,20^. Bleeding risk in patients undergoing hepatectomy is perceived to outweigh the postoperative risk of VTE by some surgeons, especially with increased volume resection^21,22^. ACCP/CHEST guidelines recommend the use of pharmacological prophylaxis that includes subcutaneous LMWH or low-dose unfractionated (LDUH) heparin or enoxaparin for borderline and high-risk donors for uncomplicated VTE prevention and treatment^11^. The median duration of heparin administration for donors at our centre in the borderline and high-risk group was 5 days (range, 1–7 days). As hypercoagulability can remain several days after hepatic resection, a longer period of prophylaxis can be desirable unless the donor develops a severe side effect related to anticoagulation, but we discontinued pharmacologic VTE prophylaxis after 5 days in the majority of the donors postoperatively. Fortunately, none of the donors had VTE at the median follow-up of 2.5 years (1 month–2 years). However, we encourage the continuation of careful monitoring for the long-term follow-up of donors in the borderline and high-risk groups.

A limitation of this study is that all the donor candidates who were evaluated were of Southeast Asian descent. A significant difference exists in the type and prevalence of hereditary thrombophilia between Asian and Western populations^23–25^. Another limitation of this study is the lack of post-hepatectomy data on PC, PS, and AT-III levels after surgery in the donors as these factors are synthesized in the liver which has reduced significantly after hepatectomy and has short half-lives, which may exaggerate already exiting hypercoagulable state. Bezaeaud *et al.* ^26^ documented the changes in coagulation after partial hepatectomy in 12 living donors. This report showed that AT-III decreased by 50% of baseline, which remained till day 5 postoperatively and coincided with a similar size decrease in PC levels^26^. PS also showed a temporary decrease in this report but returned to normal within 24 h^26^. More studies are required to clearly define the lowest cut-off level of PS and PC safe for LDLT without compromising the safety of donors.

## Conclusions

Due to thrombophilia testing directed VTE prophylaxis, VTE events were comparable in normal, borderline and high-risk thrombophilia donor groups, but more evaluations are required to determine the lower safe levels for various thrombophilia parameters including PC, PS and AT-III before surgery among living donor candidates.

## Ethical approval

The PAQSJ Institute of Medical Sciences institutional ethical review board in Gambat, Pakistan, approved this study (Reference UIN: PAQSJIMS/IRB/865).

## Consent

Written informed consent was obtained from the patients for publication and any accompanying images. A copy of the written consent is available for review by the Editor-in-Chief of this journal on request.

## Sources of funding

There is no funding received for this study.

## Author contribution

A.W.D., and A.H. conceived the idea; A.G. and S.U.D. collected the data; A.H. and A.W.D. Analyzed and interpreted the data; S.U.D., A.H., K.U., M.J.T., and H.F.U.S. did write up of the manuscript; and finally, M.J.T., A.W.D. and K.A.H. reviewed and revised the manuscript for intellectual content critically. All authors approved the final version of the manuscript.

## Conflicts of interest disclosure

There is no conflict of interest of any author.

## Research registration unique identifying number (UIN)

1. Name of the registry: Not required.
2. Unique Identifying number or registration ID: Not applicable.
3. xHyperlink to your specific registration (must be publicly accessible and will be checked):

## Guarantor author and authors critical approval

All authors have read and approved the final version of the manuscript have full access to all the data in this study and take complete responsibility for the integrity of the data and the accuracy of the data analysis.

## Data statement and availability

All the relevant study data are available from the corresponding author upon reasonable request.

## Provenance and peer-review

Not commissioned, externally peer-reviewed.

## Transparency statement

All authors affirm that this manuscript is an honest, accurate, and transparent account of the study being reported; that no important aspects of the study have been omitted; and that any discrepancies from the study as planned (and, if relevant, registered) have been explained.
